# Genetic and metabolic characterization of individual differences in liver fat accumulation in Atlantic salmon

**DOI:** 10.3389/fgene.2025.1512769

**Published:** 2025-02-13

**Authors:** Siri S. Horn, Anna K. Sonesson, Aleksei Krasnov, Muhammad L. Aslam, Borghild Hillestad, Bente Ruyter

**Affiliations:** ^1^ Nofima (Norwegian institute of Food, Fisheries and Aquaculture research), Tromsø, Norway; ^2^ SalmoBreed AS, Bergen, Norway

**Keywords:** Atlantic salmon, lipid metabolism, liver fat, heritabiity, gene expreesion

## Abstract

**Introduction:**

Lipid accumulation in the liver can negatively impact liver function and health, which is well-described for humans and other mammals, but relatively unexplored in Atlantic salmon. This study investigates the phenotypic, genetic, and transcriptomic variations related to individual differences in liver fat content within a group of slaughter-sized Atlantic salmon reared under the same conditions and fed the same feed. The objective was to increase the knowledge on liver fat deposition in farmed salmon and evaluate the potential for genetic improvement of this trait.

**Methods:**

The study involved measuring liver fat content in a group of slaughter-sized Atlantic salmon. Genetic analysis included estimating heritability and conducting genome-wide association studies (GWAS) to identify quantitative trait loci (QTLs). Transcriptomic analysis was performed to link liver fat content to gene expression, focusing on genes involved in lipid metabolic processes.

**Results:**

There was a large variation in liver fat content, ranging from 3.6% to 18.8%, with frequent occurrences of high liver fat. Livers with higher levels of fat had higher proportions of the fatty acids 16:1 n-7, 18:2 n-6, and 18:1 n-9, and less of the long-chain omega-3 fatty acids. The heritability of liver fat was estimated at 0.38, and the genetic coefficient of variation was 20%, indicating substantial potential for selective breeding to reduce liver fat deposition in Atlantic salmon. Liver fat deposition appears to be a polygenic trait, with no large QTLs detected by GWAS. Gene expression analysis linked liver fat content to numerous genes involved in lipid metabolic processes, including key transcription factors such as LXR, SREBP1, and ChREBP.

**Discussion:**

The results indicated a connection between liver fat and increased cholesterol synthesis in Atlantic salmon, with potentially harmful free cholesterol accumulation. Further, the gene expression results linked liver fat accumulation to reduced peroxisomal β-oxidation, increased conversion of carbohydrates to lipids, altered phospholipid synthesis, and possibly increased *de novo* lipogenesis. It is undetermined whether these outcomes are due to high fat levels or if they are caused by underlying metabolic differences that result in higher liver fat levels in certain individuals. Nonetheless, the results provide new insights into the metabolic profile of livers in fish with inherent differences in liver fat content.

## 1 Introduction

The liver is the central organ for metabolism and transport of lipids in Atlantic salmon. It is an important site for lipid β-oxidation (Stubhaug et al., 2005; Stubhaug et al., 2007), lipoprotein and triacylglyceride (TAG) synthesis (Kjær et al., 2008; Moya-Falcon et al., 2005; Vegusdal et al., 2005), as well as synthesis of cholesterol and bile (Cruz-Garcia et al., 2009; Leaver et al., 2008). Any imbalance in these processes can lead to lipid accumulation in the liver, which over time, can negatively impact liver function and health. Fatty liver, known as hepatic steatosis, is characterized by accumulation of lipids, primarily TAG, free fatty acids (FFA), and cholesterol (Puri et al., 2007). Triglycerides can be synthesized from FFA in the liver, and is assembled into very low-density lipoprotein (VLDL) particles for secretion. Under normal circumstances only small amounts of TAG are stored in the liver in lipid droplets (Alves-Bezerra and Cohen, 2017).

Pathological accumulation of lipids in hepatocytes is thoroughly studied in humans, and characterizes non-alcoholic fatty liver disease (NAFLD), or as recently proposed “metabolic-associated fatty liver disease” (MAFLD), which can progress from simple steatosis to end-stage liver diseases (Heeren and Scheja, 2021). It is closely related to disturbances in energy metabolism, and is linked to factors like disrupted lipid metabolism, increased cholesterol synthesis, inflammation, oxidative stress, lipotoxicity and mitochondrial dysfunction (Banini and Sanyal, 2016; Puri et al., 2007). There are several known genetic polymorphisms with strong links to liver fat accumulation in humans, including Patatin-like phospholipase domain-containing protein 3 (PNPLA3), Apolipoprotein C3 (APOC3), and Glucokinase regulator (GCKR) (Eslam et al., 2018; Namjou et al., 2019; Romeo et al., 2008).

The characterization of hepatic steatosis in Atlantic salmon remains relatively unexplored. There is currently a knowledge gap regarding what level of liver fat is unhealthy for the fish, and what metabolic factors are causing variation in the level of liver fat in Atlantic salmon. The question of whether lipid accumulation in the liver of salmon mirrors that observed in rodents and humans is still unanswered, although preliminary evidence suggests a similarity. Espe et al. (2019) have developed a fatty liver model in Atlantic salmon liver cells, which closely resembles the established mammalian model. Just as in mammals, elevated levels of liver fat in Atlantic salmon have been associated with adverse health effects and increased mortality. Dessen et al. (2020) observed a link between increased liver fat and sudden mortality of seemingly healthy Atlantic salmon in sea cages. Increased levels of liver lipids in Atlantic salmon are typically seen in feed trials involving high inclusions of vegetable oils and low levels of omega-3 fatty acids, where it coincides with reduced health and increased mortality rates (Bou et al., 2017; Torstensen et al., 2011). Hepatic steatosis is therefore considered a typical essential fatty acid deficiency symptom in salmonids, where it is characterized by a pale and swollen appearance of the liver (Ruyter and Thomassen, 1999).

Kjaer et al. (2008) showed that high dietary levels of the marine omega-3 fatty acids lowered TAG secretion from salmon hepatocytes, and lowered liver fat deposition. It is therefore assumed that salmon fed a high fish-oil diet rich in marine omega-3 fatty acids will not develop unhealthy high levels of liver fat. However, in a previous study by our group, we measured the liver fat content of ∼50 slaughter-sized Atlantic salmon fed a commercial broodstock feed, and found large variation among the fish (Horn et al., 2019). A recent study corroborates our findings, demonstrating high individual variation in total liver fat within feed groups (Hundal et al., 2022). This prompted us to investigate the genetic variation underlying this trait. The use of selective breeding programs to improve traits of commercial interest have been ongoing since the late 1960s in Atlantic salmon aquaculture (Gjedrem, 2005). It is unknown if liver fat is a heritable trait suitable for inclusion in Atlantic salmon selective breeding programs. The apparent importance of liver fat regarding fish health and robustness, together with the high mortality rate of salmon production suggest that selective breeding for this trait have the potential to improve fish welfare and reduce production costs in Atlantic salmon farming.

The specific aims of the present study were to genetically and metabolically characterize individual differences in liver fat content in Atlantic salmon through gene expression analysis, quantitative genetic analysis and genome-wide association analysis in order to increase the knowledge on liver fat deposition in slaughter-sized farmed salmon and evaluate the potential for genetic improvement of the trait.

## 2 Materials and methods

### 2.1 Fish populations and recordings

The fish studied originated from families and documentation groups from the 2014 years-class of the SalmoBreed Atlantic salmon strain of Benchmark Genetics Norway AS, former SalmoBreed AS. The fish were transferred to sea at a mean weight of 0.1 kg, and slaughtered approximately 12 months later, at a mean weight of 3.6 kg. The fish were fed a commercial broodstock feed from Skretting AS with a relatively high fish oil content, where the sum of EPA and DHA comprised 6.2% of feed, and 17% of total fat in feed (Supplementary Material S1). All the fish were reared under the same conditions and were fasted 13–14 days prior to slaughter.

At slaughter, sex was determined visually by inspection of the gonads, body and liver weight was recorded, and liver color, used as an indicator of the degree of fatty liver, was determined visually on a scale of 1 (darkest color, i.e., healthy liver) to 5 (lightest color i.e., highest amount of fat) (Mørkøre et al., 2012). For the data analysis, liver color scale was reversed to allow for a more intuitive presentation of results. Hepatosomatic index (HSI) was calculated as (liver weight/body weight)*100. Liver tissue samples for RNA-sequencing were taken from each individual fish at harvest, immediately frozen in liquid nitrogen, and subsequently stored at – 70°C. Liver samples for lipid and fatty acid analysis were collected at harvest, frozen and stored at −20°C.

The fish material in the current study was part of the dataset described in Horn et al. (2019), where the fish were analyzed for skeletal muscle lipid and fatty acid composition. In the current study, livers of 610 fish were analyzed for lipid level (grams lipid per 100 g liver), and liver tissue of 48 fish was selected for RNA-sequencing and liver fatty acid analysis. The selected 48 individuals were of similar bodyweight (3.3–3.9 kg) in order to minimize the effects of size, and had skeletal muscle fat level within the normal range (16%–25%) to avoid outlier individuals regarding lipid deposition in muscle. The selected individuals were 55% males and 45% females and originated from 39 sires and 48 dams.

### 2.2 Lipid and fatty acid analysis

Total lipids were extracted from homogenized liver samples of individual fish, according to the Folch method (Folch et al., 1957). Using the chloroform-methanol phase, fatty acid composition was analyzed following the method described by Mason and Waller (1964). The extract was dried briefly under nitrogen gas and residual lipid extract was trans-methylated overnight with 2′,2′-dimethoxypropane, methanolic-HCl, and benzene at room temperature. The methyl esters formed were separated in a gas chromatograph (Hewlett Packard 6,890; HP, Wilmington, DE, USA) with a split injector, using an SGE BPX70 capillary column (length 60 m, internal diameter 0.25 mm, and film thickness 0.25 μm; SGE Analytical Science, Milton Keynes, UK) and a flame ionization detector. The results were analyzed using HP Chem Station software. The carrier gas was helium, and the injector and detector temperatures were both 270°C. The oven temperature was raised from 50°C to 170°C at the rate of 4°C/min, and then raised to 200°C at a rate of 0.5°C/min and finally to 240°C at 10°C/min. Individual fatty acid methyl esters were identified by reference to well-characterized standards (23:0). The content of each fatty acid was expressed as a percentage of the total amount of fatty acids in the analyzed sample. Liver fat was expressed in %, and calculated by dividing the weight of total lipids in the liver sample by the total weight of the liver sample.

### 2.3 Gene expression analysis

Total RNA was extracted from liver tissues of 48 fish using the PureLink Pro 96 RNA Purification Kit (Invitrogen), according to the manufacturer’s instruction. RNA was treated with PureLink On-Column DNase Digestion (Invitrogen) to remove any contaminating DNA. Samples were shipped to The Norwegian High-Throughput Sequencing Centre, where the mRNA library preparation and sequencing of transcripts were performed using standard protocols (www.illumina.com). Samples were sequenced on an Illumina HiSeq platform as paired-end 151 bp reads.

Processing of reads, alignment and annotation was performed according to Moghadam et al. (2017). Expression data were normalized via the median of the geometric means of fragment counts across all sample (Anders and Huber, 2010). Cufflinks and Cuffdiff were used to estimate the expression abundances of the assembled genes and transcripts (Trapnell et al., 2010). Gene expression data were normalized by calculating the aligned fragments per kilobase per million mapped fragments (FPKM). Normalized gene expression data were log2 transformed prior to the statistical analysis.

Trait-associated genes were defined by using linear regression analysis, testing for an association between the continuous trait and mRNA expression. The liver content of fat (%) was considered the response variable and each individual gene expression an explanatory variable in the model. As suggested by Seo et al. (2016), univariate analyses were carried out for each trait. The following general linear mixed model was fitted:

$${\text{Trait}}_{i}=\beta_{0}+\beta_{1}{\text{Expression}}_{1i}+\beta_{m}{\text{Sex}}_{\text{mi}}+\beta_{p}{\text{Family}}_{\text{pi}}+\varepsilon_{i},\varepsilon_{i}\;∼\;N\;\left(0,\sigma^{2}\right)$$

where *i* represents the individuals, *Expression*_*i*_ indicates the normalized gene expression value. *Trait*_*i*_ represents the trait liver fat %. Covariates: Sex_mi_ represents the fixed effect of sex (male or female), and Family_pi_ represents the random effect of family (1–48). Genes were considered significantly associated with the trait when the p-value of the regression coefficient was <0.05. Genes of interest are presented with their regression coefficient (Figures 6–8). All significant genes are presented in Supplementary Material S2.

### 2.4 Enrichment analysis

A search for enriched GO classes and KEGG pathways in the list of 1,872 trait-associated genes was performed by counting of genes among the 1,781 trait-associated genes that passed quality control. Enrichment was assessed with Yates’ corrected chi square test (p < 0.05). Terms with less than five genes were not taken into consideration.

### 2.5 Genotyping

The fish were genotyped using a customized ∼57 K axiom Affymetrix SNP Genotyping Array (NOFSAL02). From the initial ∼57 K SNPs quality filtering was performed using criteria of call rate >0.9, minor allele frequencies >0.02, and Hardy-Weinberg equilibrium correlation p-value >0.001. A total of 52,925 SNPs passed quality control filtering and were used to compute the genomic relationship matrix (GRM) and in the GWAS.

### 2.6 Estimation of genetic parameters

Variance components were estimated from a univariate restricted maximum likelihood (GREML) analyses, with “--reml” function in GCTA program (Yang et al., 2014) with the following model on a total of 610 fish:

Y=µ+Xβ+Zu+e

where Y is the vector of phenotypic observations of all individuals; µ is overall mean; β is the vector of fixed effect of sex; X is the corresponding incidence matrix; u is the vector of additive genetic effects with $u∼N\left(0,G\sigma_{u}^{2}\right)$ , where $\sigma_{u}^{2}$ is the additive genetic variance, and Z the corresponding incidence matrix; and e is the vector of random residual effects with $e∼N\left(0,I\sigma_{e}^{2}\right)$ . G is a genomic relationship matrix (GRM) which was computed according to VanRaden (2008) as $\frac{ZZ^{\prime }}{2*\sum_{i=1}^{Nsnp}p_{i}\left(1-p_{i}\right)\;}$ ; where $p_{i}$ is the allele frequency of second allele and Nsnp is the total number of SNP markers.

Heritability (narrow sense) was estimated as the ratio of additive genetic variance to total phenotypic variance. Genetic correlations between pairs of phenotypes were estimated with “--reml-bivar” function in GCTA, using a bivariate model - a direct extension of the above univariate model where the vectors were extended to matrices (Lee et al., 2012).

The Coefficient of variation was calculated as the ratio of the standard deviation to the mean.

**FIGURE 1:**
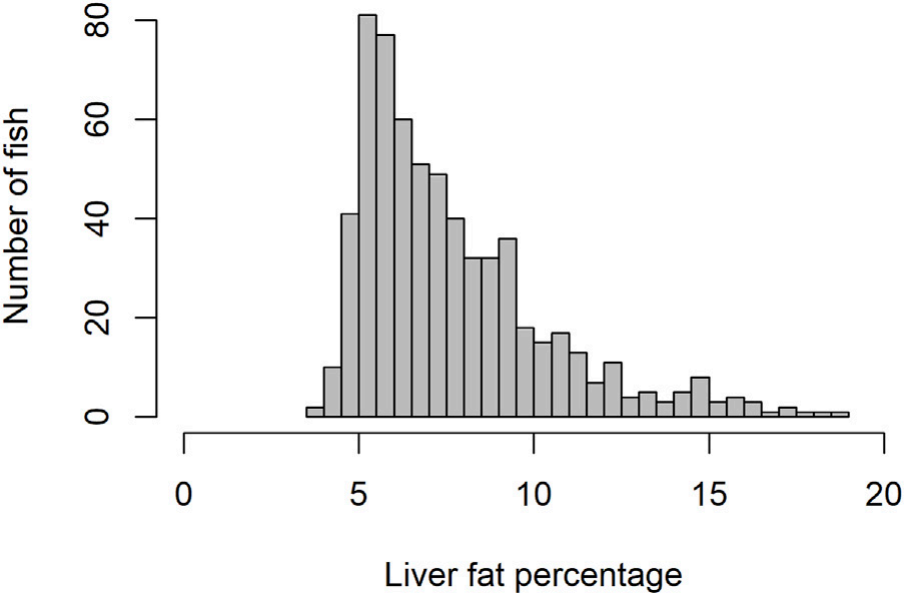
Histogram showing distribution of liver fat percentage.

Due to the presence of negative skewness in phenotype distribution (Figure 1), log transformation for the liver fat phenotype was tested. However, results regarding genetic parameters and GWAS did not differ significantly when analyzed with non-log vs log-transformed phenotypes. Results published were based on non-log transformed data.

### 2.7 GWAS

Genome wide association analysis was performed using the following linear mixed animal model implemented in GCTA program with the “–mlma-loco” function (Yang et al., 2014).

y=μ+Xb+Sα+Zu+e

where y is a vector of n = 610 trait records, μ is an overall mean; b is a vector of fixed effects including (phenotypic) sex and 2 principal components as covariates; X is the incidence matrix for the effects contained in b ; S is the incidence matrix for SNP containing marker genotypes coded as 0=AA,1=AB|BA,2=BB , α is the allele substitution effect of each SNP, Z is the incidence matrix of genotyped individuals, u is the vector of additive genetic effects with $u∼N\left(0,G\sigma_{u}^{2}\right)$ , where $\sigma_{u}^{2}$ is the additive genetic variance, and e is the vector of random residual effects with $e∼N\left(0,I\sigma_{e}^{2}\right)$ .

The trait SNP association was considered significant using two thresholds: i) genome-wide significance with stringent level and ii) chromosome-wide significance with relatively less stringent. SNPs were considered genome wide significant when they exceeded the Bonferroni threshold for multiple testing (alpha = 0.05) of 0.05/tg, where tg = 52,925 (total number of SNPs genome-wide). SNPs were considered chromosome-wide significant when Bonferroni threshold for multiple testing surpassed (alpha = 0.05) 0.05/tc, where tc = 1825 (average number of SNPs per chromosome). The obtained genome-wide significant threshold used in of this study was $p\leq 9.4\times 10^{-7}$ which is equivalent to $-\log_{10}\left(p\right)=6.0$ , while chromosome-wide significant threshold was opted to be $p\leq 2.7\times 10^{-5}$ which is equal to $-\log_{10}\left(p\right)=4.6$ .

A quantile-quantile (Q-Q plot) plot with distribution of observed vs expected p-values was checked, and the Inflation factor (lambda, λ) was calculated using following equation:

$$lambda\left(\lambda \right)=\frac{median\left(\chi^{2}\right)}{0.455}$$

## 3 Results

### 3.1 Liver fat content and fatty acid composition

**FIGURE 2:**
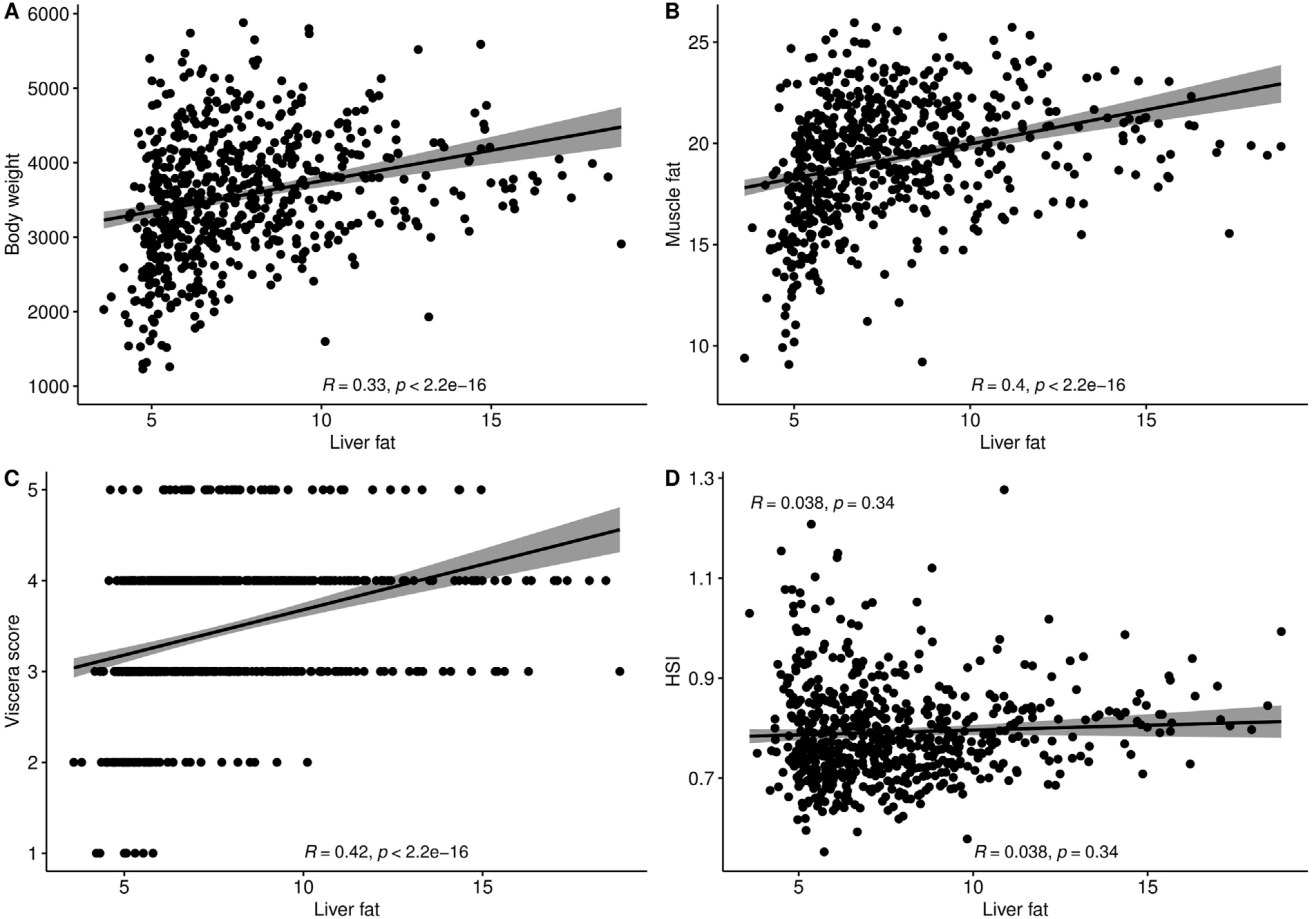
Phenotypic correlation plots and Spearman correlation coefficients of liver fat (%) with **(A)** Body weight (g), **(B)** Muscle fat (%), **(C)** Viscera score, and **(D)** Hepatosomatic index (HSI).

The level of fat in livers of the studied fish displayed large variation, ranging from 3.6% to 18.8%, with a mean of 7.7%. The trait did not display normal distribution. There seemed to be a minimum level of liver fat of about 4%, and only 16% of the 634 fish had a liver fat content higher than 10% (Figure 1). There were significant positive phenotypic correlations between liver fat and both body weight and muscle fat, but the correlations were relatively low (Figure 2). Fish with low liver fat content displayed large variation in body weight and muscle fat, while fish with more than 10% liver fat all had medium to high muscle fat level. There was no significant correlation between liver fat percentage and hepatosomatic index (HSI) (Figure 2).

The 48 fish selected for RNA sequencing and analysis of liver fatty acid composition showed that within a 600 g body weight range, the liver fat percentage varied greatly, from 5% to 19% (Table 1). The major fatty acids in the liver, constituting more than 47% of liver fatty acids, were oleic acid (18:1n-9; 22%), DHA (22:6n-3; 14%) and palmitic acid (16:0; 11%). There was a small variation in the EPA relative content of liver (5%–8%), compared to a relatively large variation in DHA relative content (6%–20%). This is opposite to what we found in the muscle of the same fish material, where the DHA relative content was more stable, and EPA varied significantly (Horn et al., 2019). The mean DHA relative content was also higher in liver compared to muscle, although the quantitative content of DHA was higher in muscle due to the higher fat content of muscle.

**TABLE 1:** Descriptive statistics of all fish, and fish in the gene expression analysis (n = 48).

	n	Mean	SD	Min	Max
All fish					
Body weight (kg)	634	3.56	0.83	1.23	5.88
Muscle fat (%)	634	19.2	3.0	9.1	26.0
Liver fat (%)	634	7.6	2.7	3.6	18.8
HSI	634	0.8	0.1	0.6	1.3
Fish selected for gene expression analysis					
Body weight (kg)	48	3.64	0.16	3.33	3.89
Muscle fat (%)	48	19.8	2.1	16.2	25.3
Liver fat (%)	48	9.1	0.5	5.1	18.8
Liver fatty acids (%)					
16:0	48	11.1	1.9	7.6	14.3
16:1 n-7	48	2.3	0.7	1.4	3.7
18:0	48	5.2	0.6	4.0	6.4
18:1 n-9	48	22.4	5.6	12.6	33.1
18:2 n-6	48	5.6	0.9	3.6	7.9
18:3 n-3	48	2.2	0.4	1.4	3.0
20:5 n-3 (EPA)	48	6.5	0.7	4.8	8.0
22:5 n-3	48	3.5	0.5	2.1	4.3
22:6 n-3 (DHA)	48	13.8	4.0	6.5	20.4

N, number of fish; SD, Standard deviation; Min, minimum value; Max, maximum value.

**FIGURE 3:**
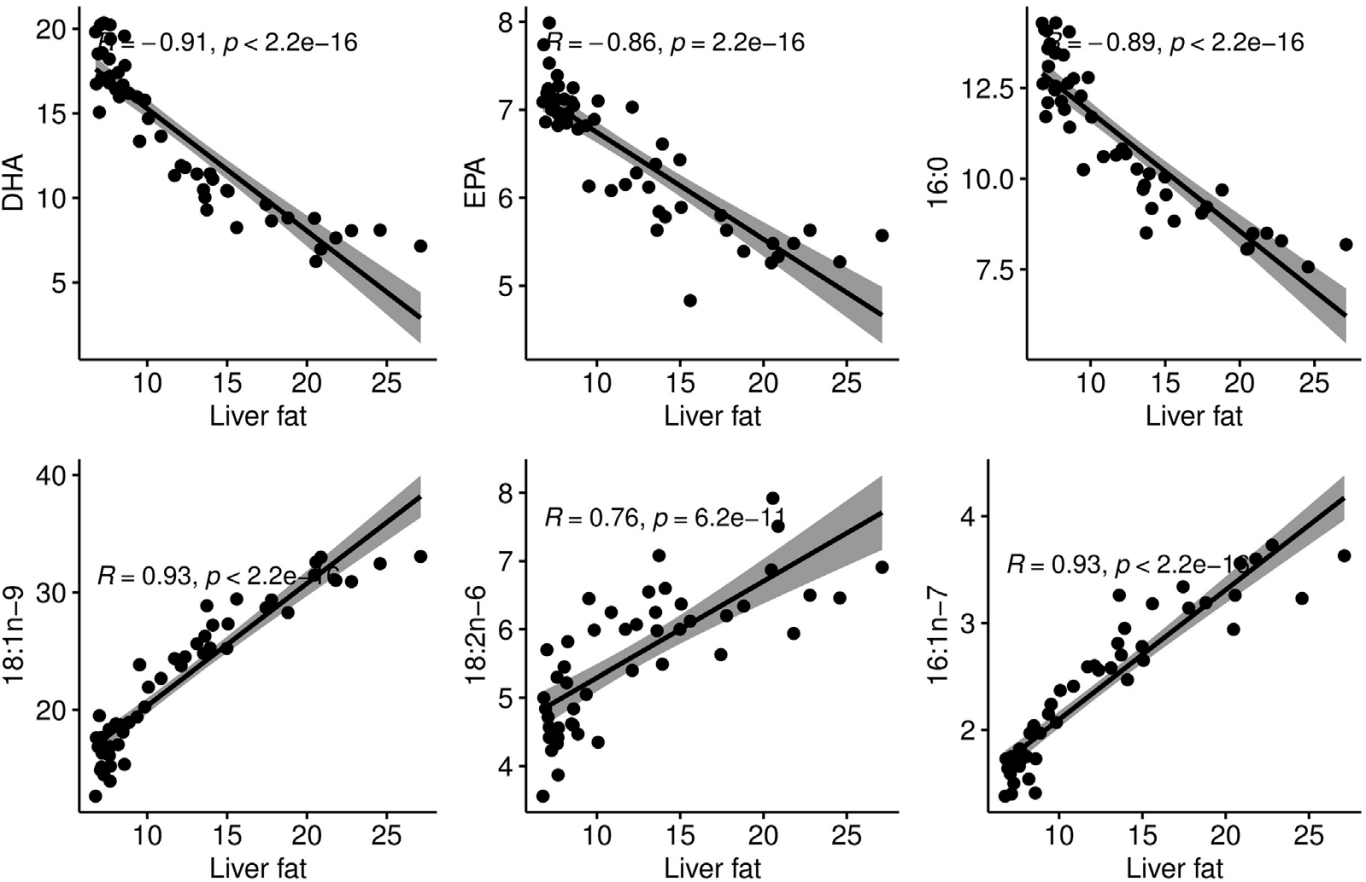
Correlation plots between liver fat percentage and proportional content of individual fatty acids in the liver.

The phenotypic correlations between liver fat and individual fatty acids showed that in this group of fish of similar size, the fattier livers had a significantly higher percentage of 16:1 n-7, 18:2n-6 and 18:1n-9. The percentage of the marine omega-3 fatty acids EPA and DHA decreased significantly with increasing liver fat (Figure 3). Interestingly, palmitic acid (16:0), the most common saturated fatty acid in the body, followed the same pattern as EPA and DHA.

### 3.2 Heritability of liver fat

This study is the first to report the heritability of liver fat content in Atlantic salmon. The heritability estimate for liver fat content was 0.38, with a standard error of 0.07 (Table 2). The genetic standard deviation was 1.6% fat, corresponding to a genetic coefficient of variation of approximately 20%. The genetic correlations between liver fat and the other traits were similar to the phenotypic correlations, although the genetic correlation with muscle fat was stronger than with body weight or viscera score (Table 3). The genetic correlation between liver fat and liver score was 0.7, which was slightly stronger than the phenotypic correlation.

**TABLE 2:** Genetic parameters of liver fat.

	Variance (σ^2^)	SD (σ)
Genetic	2.59 ± 0.58	1.61
Residual	4.19 ± 0.43	2.05
Phenotypic	6.78 ± 0.44	2.60
**H** ^**2**^	**0.38 ± 0.07**	

SD, standard deviation; H^2^, heritability.

**TABLE 3:** Genetic correlations between liver fat and lipid-related traits.

	H^2^	rG liver fat
Muscle fat	0.43 (0.08)	0.37 (0.14)
Body weight	0.58 (0.07)	0.31 (0.12)
Liver score	0.28 (0.07)	0.70 (0.12)
HSI	0.19 (0.07)	0.25 (0.20)
Viscera score	0.28 (0.07)	0.28 (0.17)

Standard errors in brackets. H^2^, heritability; HSI, hepatosomatic index; rG Liver fat, genetic correlation with liver fat.

### 3.3 Genome wide association study

**FIGURE 4:**
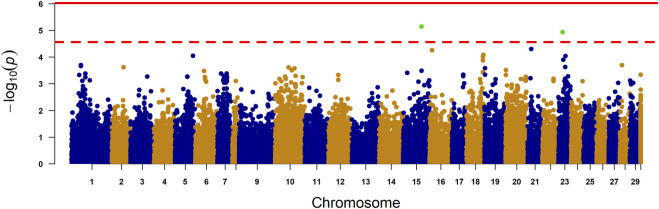
Manhattan plot for liver fat content. The X-axis represents the chromosomes, and the Y-axis shows the–log 10 (p-value). The solid line represents Bonferroni-corrected genome-wide significant threshold. The dashed line represents the suggestive chromosome-wide significance threshold. The final yellow column is for SNPs with unknown genome placement. Lambda = 1.18.

There were no SNPs surpassing the Bonferroni corrected genome-wide significance threshold, but two SNPs did surpass the chromosome-wide threshold (Figure 4). These SNPs were AX-87183264, located on chromosome 15 (p-value 7.23e-06), and AX-98317599, located on chromosome 23 (p-value 1.17e-05). We used functional annotation data (Assembly Ssal_v3.1 (GCF_905237065.1)) to perform a detailed investigation of genes located within approx. ±200 kb of the 10 most significant SNPs to identify candidate genes that may influence lipid deposition in the liver. The top 10 SNPs and the candidate genes detected within this specified region are detailed in Supplementary Material S3.

No known genes of direct relevance were detected underlying the QTL region on chromosomes 23 and 16. One of the genes located closest to the top SNP on ssa15 is *Mechanistic target of rapamycin kinase (mTOR). mTOR* regulates lipid metabolism and has been suggested as a potential new target in NAFLD (Feng et al., 2022). In salmonids, research has shown that Rainbow trout genetically selected for greater muscle fat content display increased activation of liver TOR signaling and lipogenic gene expression (Skiba-Cassy et al., 2009). Within a 120 kb region of the top SNP on chromosome 21, we identified the gene *Glycerol-3-phosphate dehydrogenase (GPDH)*. This enzyme is crucial for linking carbohydrate metabolism to lipid metabolism. Our findings revealed a significant association between the expression of *GPDH* and liver fat deposition (see Supplementary Material S2). Although the expression was attributed to a copy of the gene located on a different chromosome (ssa12), the salmon genome contains multiple copies of the *GPDH* gene. This redundancy may lead to challenges in accurately differentiating or assigning the transcribed copies to their respective genes within the transcriptome. Consequently, the observed gene expression results may reflect genetic variation in the gene located on ssa21. Three of the top 10 SNPs were located on ssa18 in the region from 64,700,815 to 65,948,775 bp. We identified one gene in the nearby region known to be linked to lipid deposition in the liver: *Perforin 1*. *Perforin 1* has been linked to NAFLD in both humans and mice through its role in regulating the immune system. One study showed that perforin-deficient mice exhibit increased lipid accumulation in the liver and more liver inflammation when subjected to a high-fat diet (Wang et al., 2020). Indeed, the expression of two *perforin-1-like* genes was negatively associated with liver fat deposition in our study (Supplementary Material S2). Within 20 kb of the top SNP on ssa05 is the gene for *Hormone-sensitive lipase (HSL). HSL* is a key enzyme involved in the hydrolysis of triglycerides into free fatty acids and glycerol. Studies have shown that individuals with hereditary deficiency of *HSL* develop fatty liver (Xia et al., 2017). The gene expression of *HSL* was not significantly associated with liver fat in our study.

### 3.4 Gene expression associations with liver fat

The gene expression association analysis identified 1781 genes as significantly associated with liver fat. In total, 74 KEGG/GO pathways were enriched (Supplementary Material S4). We limit this paper to metabolic processes involved in lipid metabolism and/or linked to fatty liver in mammals. This includes cholesterol biosynthesis, *de novo* lipogenesis (DNL), triacylglyceride (TAG) synthesis, phospholipid synthesis and beta-oxidation. Genes of interest are presented in Figures 6–8 with their regression coefficients. All significant genes are presented in Supplementary Material S2.

#### 3.4.1 Cholesterol biosynthesis

**FIGURE 5:**
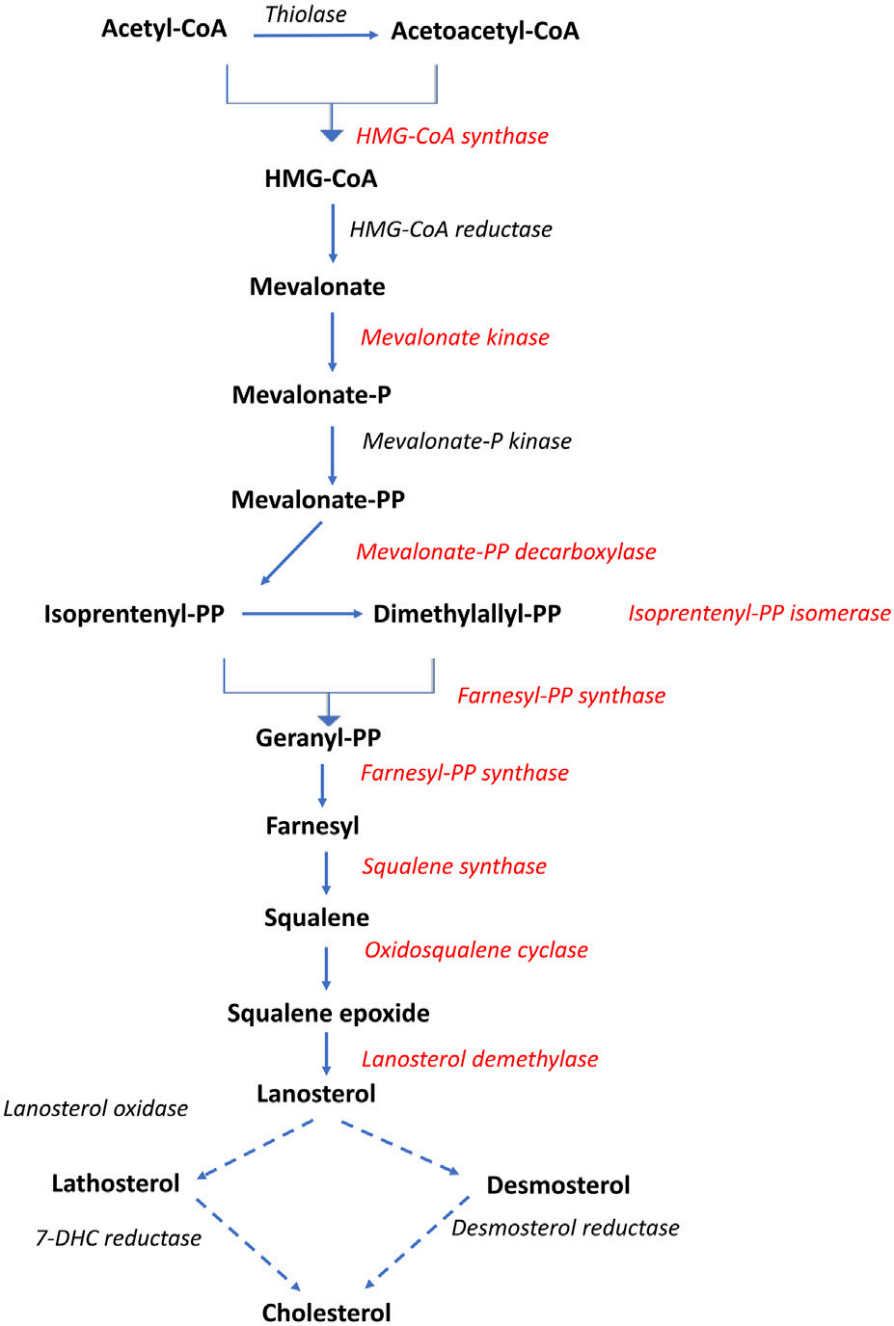
Overview of the cholesterol biosynthetic pathway (Mevalonate pathway). Dotted arrows indicate that multiple enzymatic steps are involved. Red text indicates gene expression positively associated with liver fat. Adapted after Liscum, 2008.

**FIGURE 6:**
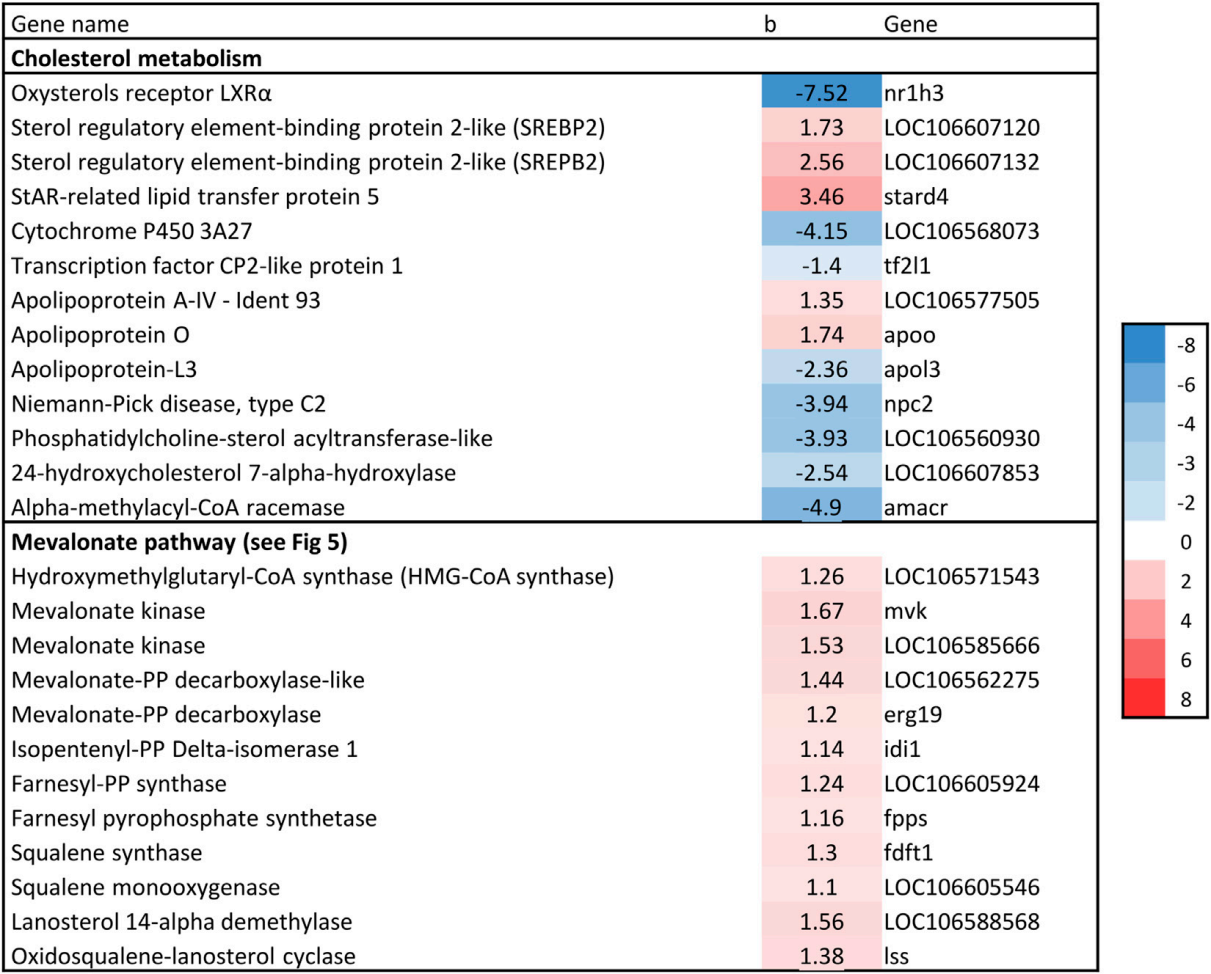
Association between liver fat percentage and hepatic expression of genes involved in cholesterol biosynthesis, b = linear regression coefficient. Color intensity indicate strength of association.

Nine genes directly involved in the biosynthesis of cholesterol from acetyl-CoA, known as the mevalonate pathway, were significantly associated with liver fat content. This included the rate-limiting enzyme in cholesterol biosynthesis, *hydroxymethyl-glutaryl-CoA* (HMG-CoA) *reductase*, as well as *squalene synthase*, which catalyzes the first committed step in cholesterol formation (Liscum, 2008). All nine genes had a positive association with liver fat, indicating that fish with higher liver fat content had a higher production of cholesterol (Figures 5, 6).

#### 3.4.2 Hepatic *de novo* lipogenesis

**FIGURE 7:**
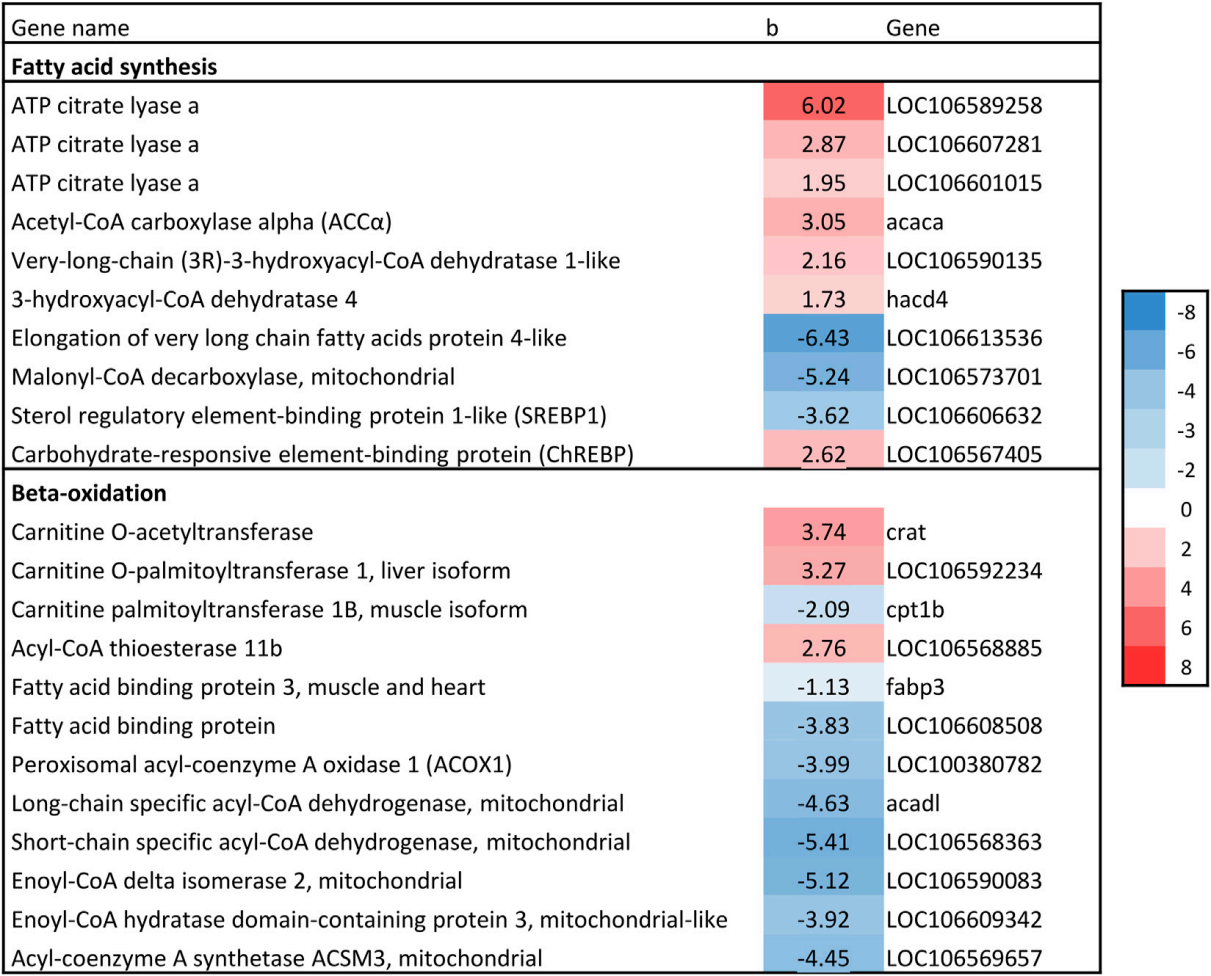
Association between liver fat percentage and hepatic expression of genes involved in fatty acid synthesis and beta-oxidation. b = linear regression coefficient. Color intensity indicate strength of association.

Two of the major regulators that promotes DNL, LXRα and SREBP1c, were negatively associated with increasing liver fat. However, expression of ChREBP, another major regulator of lipogenesis, as well as three isoforms of *ATP citrate lyase* (ACLY) were positively associated with liver fat (Figure 7). ACLY is a key lipogenic enzyme that links carbohydrate and lipid metabolism by catalyzing production of acetyl-CoA from citrate. Expression of ACCα was also positively associated with liver fat (Figure 7). ACCα produces malonyl-CoA and is regarded as the pace-setting enzyme for fatty acid synthesis, and is induced by ChREBP (Sul and Smith, 2008). In addition, *Malonyl-CoA decarboxylase*, which catalyzes the opposite reaction of ACCα, was negatively associated with liver fat (Figure 7).

#### 3.4.3 Beta-oxidation

Once fatty acids are activated to acyl-CoAs, they can be esterified by GPAT and enter the glycerol phosphate pathway, or they can be converted to acyl-carnitines by carnitine palmitoyltransferase-1 (CPT1) and then enter the mitochondrion for β-oxidation. Two CPT1 genes were associated with liver fat, but in opposite directions (*carnitine O-palmitoyltransferase 1 (liver isoform)* and *carnitine palmitoyltransferase 1B (muscle isoform),* Figure 7). The expression of some genes directly involved in fatty acid beta-oxidation were negatively associated with liver fat. This *included Long-* and *short-chain specific acyl-CoA dehydrogenase* which catalyze the first step of mitochondrial fatty acid beta-oxidation, and *Enoyl-CoA delta isomerase 2*, which is required for degradation of unsaturated fatty acid (Schulz, 2008) (Figure 7). This also included *Peroxisomal acyl-coenzyme A oxidase* (ACOX1), a rate-limiting enzyme in peroxisomal fatty acid β-oxidation.

#### 3.4.4 Glycerol phosphate pathway - triglyceride and phospholipid synthesis

**FIGURE 8:**
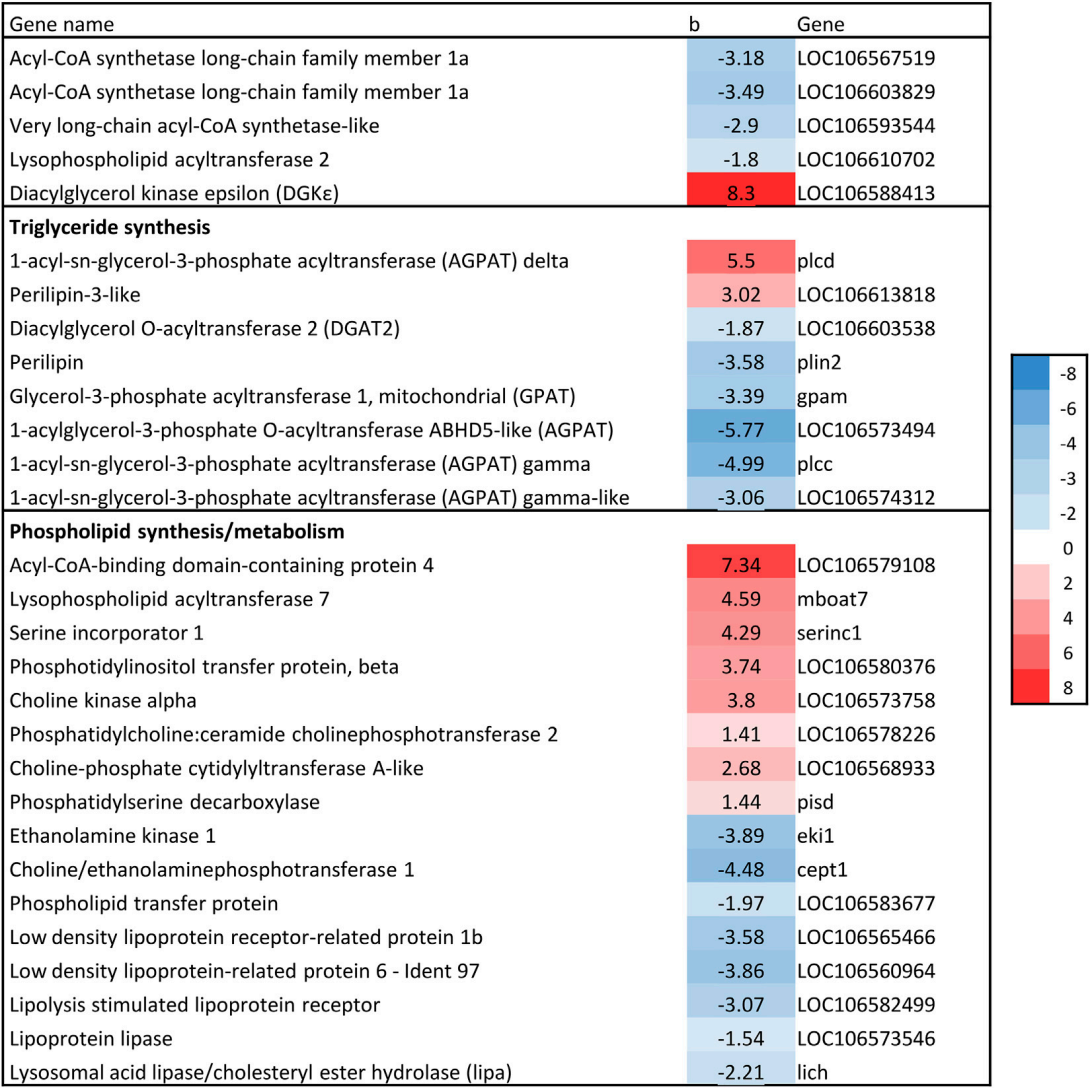
Association between liver fat percentage and hepatic expression of genes involved in Glycerol phosphate pathway. b = linear regression coefficient. p = p-value of association. Color intensity indicate strength of association.

The first committed step in TAG synthesis via the glycerol phosphate pathway is mediated by glycerol-3-phosphate acyltransferase (GPAT) enzymes (Sul and Smith, 2008), and produce lysophosphatidic acid (LPA). GPAT gene expression was negatively associated with liver fat in the current study, which is in accordance with the expression of SREBP-1, considering that SREBP-1c induces the expression of GPAT1 (Karasawa et al., 2019). Further in the glycerol phosphate pathway, an additional fatty acid is transferred to LPA by the family of *1-acylglycerol-3-phosphate acyltransferase* (AGPAT) enzymes to produce phosphatidate (PA) (Sul and Smith, 2008). Four AGPAT genes were significantly associated with liver fat, three of which had a negative association (Figure 8). The final formation of TAG can be catalyzed by several different enzymes, including *diacylglycerol:acyl-CoA acyltransferase* (DGAT). DGAT expression was negatively associated with liver fat content (Figure 8). Thus, the formation of both LPA, PA and TAG seemed to be decreasing with increasing liver fat.

The other branch of the glycerol phosphate pathway is the synthesis of phospholipids (PL). Several genes involved in PL synthesis and metabolism were significantly associated with liver fat (Figure 8). There was a positive association between liver fat and expression of genes catalyzing three steps of phosphatidylcholine (PC) synthesis from choline, indicating increased conversion of choline to PC in fish with higher liver fat accumulation. Also, lipoprotein receptors as well as *phospholipid transfer protein* were negatively associated with liver fat. All these genes are involved in processes that are likely to alter PL composition.

## 4 Discussion

In this study, we investigated the genetic and transcriptomic variation related to individual differences in liver fat content within a group of slaughter-sized Atlantic salmon reared under the same conditions and fed the same diet. Environmental factors that likely contribute to variation in liver lipid deposition include individual differences in feed intake, swimming activity, and stress or disease burden. However, discussions on these environmental factors are outside the scope of this paper.

### 4.1 Fat content of livers of Atlantic salmon

There are few published reports on accurate measurements of liver fat content of slaughter-sized Atlantic salmon, but previous studies have reported liver fat levels between 5 and 7% for slaughter sized Atlantic salmon fed a medium to high fish-oil feed (Bou et al., 2017; Dessen et al., 2020; Hundal et al., 2022; Ruyter et al., 2006), which is in line with the mean liver fat content of 7.7% in the current study. Based on our results it appears that 4% fat is the lower physiological limit in livers of slaughter-sized Atlantic salmon.

As the fish in the current study were fed a commercial broodstock feed with a relatively high fish-oil content, we would not expect unhealthy high levels of liver fat, because fatty livers have in literature mainly been observed as a consequence of insufficient dietary levels of EPA and DHA in Atlantic salmon (Bransden et al., 2003; Torstensen et al., 2011). However, 37% of the fish were given the second worst liver color score, which implies that there was a high number of fish with livers that appeared pale and fatty, and the chemical analysis revealed that 8% of the fish had liver fat content above 12%. Liver fat of 12% was recently reported in fish fed insufficient EPA + DHA and showed impaired liver metabolic health (Hundal et al., 2022). Thus, it appears that even in Atlantic salmon fed a relatively high fish-oil diet, high levels of liver fat accumulation occurs frequently. However, there is very limited knowledge on the possible health implications of this.

There was no correlation between liver fat % and hepatosomatic index (HSI) (Figure 2), which shows that HSI is not a good indicator of the level of liver fat. This is likely due to the fact that lipids weigh less than protein, so an increase in lipids will not give a corresponding increase in weight. To our knowledge, this is the first time data on HSI and liver fat percentage has been reported on a large number of Atlantic salmon of slaughter size.

### 4.2 Possibilities for selective breeding to reduce liver lipid accumulation

A heritability of 0.38, along with an additive genetic standard deviation of 1.6% fat, shows that there is a great potential for selection for lower fat deposition in liver in Atlantic salmon. A genetic standard deviation of 1.6% corresponds to a genetic coefficient of variation of approximately 20%, which is considered moderate to high and suggests that there is substantial genetic variability within the population. In the context of a breeding program, a CV of 20% implies that selective breeding can effectively increase or decrease liver fat content, depending on the breeding goals. The relatively low genetic correlation with body weight (0.33) implies that selection for lower liver fat can be implemented in selective breeding programs without compromising heavily on growth using selection index theory (Hazel, 1943). Reducing liver fat through selective breeding could lead to healthier fish with improved liver function and overall robustness, which are likely to have better growth rates and feed efficiency, contributing to more sustainable farming practices.

While the dataset was limited in size, the high-precision phenotype is a strength of the estimates obtained. To the best of our knowledge this is the first report of heritability of liver fat in Atlantic salmon, although genotype-specific responses in liver lipid metabolism in Atlantic salmon have previously been shown when fish oil has been replaced by vegetable oil in the feed (Morais et al., 2011a; Morais et al., 2011b). No reports of heritability of liver fat could be found for other fish species, but it has been reported to be a heritable trait in other animal species previously; such as duck (h2 = 0.07–0.14), chicken (h2 = 0.36–0.43) and mice (h2 = 0.22) (Liang et al., 2015; Marie-Etancelin et al., 2014; Minkina et al., 2012). In human, the heritability estimates of NAFLD generally range from 0.2 to 0.7, depending on the study design, ethnicity, and the methodology used (Sookoian and Pirola, 2017).

More than five genes, including PNPLA3, TM6SF2, GCKR, MBOAT7, and HSD17B13, are linked to NAFLD in humans [reviewed in Eslam et al. (2018)]. It is plausible that genetic variants in Atlantic salmon also affect liver fat accumulation. However, our GWAS detected no significant signals. This suggests liver fat in Atlantic salmon is a polygenic trait, with many genes or variants having small effects. Despite testing various models and SNP quality control thresholds, the results remained unchanged. SNP effect sizes have not been published in the aforementioned human studies, although two studies the risk allele effect size for PLPLA3 as a 1.5% increase in hepatic TAG content per allele (Romeo et al., 2008), and a one-unit increase in NAFLD severity score per allele (Namjou et al., 2019). Another reason for the lack of strong QTL signals could be the limited sample size of fewer than 700 fish, which may be insufficient to detect small effect sizes. Future GWAS should include more phenotyped and genotyped fish to increase analysis power and detect QTL signals from regions with small effect sizes. We were able to detect four candidate genes for future validation, two of which were also detected in the gene expression results, but we do not consider them strong candidates based on our study alone.

A major obstacle for implementation of liver fat in breeding programs is the necessity of phenotypic recording across a large number of individuals. Chemical analysis of lipid content, though accurate, is time-consuming and costly. Thus, liver score, a visual assessment of liver color, is often used as an indicator of fatty liver. In this study, we compared liver score and chemical measurements of liver fat. The phenotypic correlation between liver fat and liver score was 0.5 (p < 0.0001), indicating low predictive power. Further, the genetic correlation between these two traits was 0.7 (Table 2). The “break-even” genetic correlation between alternative and reference methods used for genetic validation of a method, is traditionally set at 0.70–0.80 (Mulder et al., 2006; Robertson, 1959). Therefore, liver score is not recommended for genetic analyses or applications requiring high accuracy. This highlights the need for reliable, rapid methods to measure liver fat. A recent study demonstrated hyperspectral imaging as a high-throughput method with high predictive accuracy (Ortega et al., 2024). Other methods like NIR and Raman spectroscopy, developed for fillet lipid content, could potentially be adapted for liver fat measurement.

Selection for reduced liver fat should be considered in the context of feed, as gene-environment interactions may cause the best performers on one feed to rank lower on another. Although these gene-by-feed interactions have not been studied, our results imply a potential to select fish that are better metabolically adapted to the high-energy feeds currently used in salmon farming. Additionally, the results of this study have implications for salmon feed research; given the large genetic variation in liver fat deposition, nutritional studies examining liver fat as a response variable should consider the genetic background of the fish to ensure that feed trial results are not biased by unbalanced genetic material or family effects in feed groups.

### 4.3 Metabolic profile of high-fat livers

The positive correlations (both phenotypic and genetic) between liver fat and the lipid deposits of muscle (rg = 0.37) and viscera (rg = 0.28) indicate that fish with higher liver fat deposition tend to have higher overall body fat deposition. However, the relatively weak correlations suggest that fish with the highest liver fat do not necessarily have the highest visceral and/or muscle fat (as shown in Figure 2). This points to at least a partially independent regulation of these lipid deposits. As the muscle is the main lipid storage in Atlantic salmon (Aursand et al., 1994), relatively large amounts of lipids can be safely stored there. Fish that deposit lipids in the liver rather than muscle may therefore have a disturbed lipid metabolism and pattern of lipid deposition. The gene expression results of the current study further support this and point to specific metabolic processes involved in liver lipid accumulation in Atlantic salmon.

The expression of numerous genes involved in lipid metabolic processes were associated with liver fat content of Atlantic salmon in the current study. This included fatty acid synthesis, fatty acid beta-oxidation, cholesterol biosynthesis, and phospholipid synthesis, as well as some of the main transcription factors regulating lipid metabolism. It is not possible to determine which of these results are consequences of a high fat level, and which are underlying metabolic differences causing higher liver fat levels in certain individuals. The results do, however, provide new knowledge about the metabolic “picture” in livers of fish with inherent differences in liver fat content. It should be noted that the fish in the current study were subjected to a 2-week fasting period before slaughter, a standard practice in commercial salmon farming. Therefore, the associations proposed here are specific to these conditions, and represent the metabolic picture in a fasted state.

### 4.4 Increased synthesis and reduced clearance of cholesterol

Increased liver fat accumulation paralleled reduced expression of the major transcription factors LXR and SREBP1, and increased expression of SREBP2. The Liver X receptors are key metabolic regulators that control cholesterol and fatty acid homeostasis, as well as modulate inflammatory and immune pathways in mammals (Hertzel et al., 2008; Liscum, 2008). SREBP-1 and SREBP-2 are major regulators of fatty acid and cholesterol biosynthetic genes, respectively. cDNAs for LXR, SREBP-1 and SREBP-2 have been characterised in Atlantic salmon (Cruz-Garcia et al., 2009; Minghetti et al., 2011). Atlantic salmon express a single LXR gene that is most similar to mammalian LXRα (Cruz-Garcia et al., 2009). SREBP and LXR interact in the regulation of a range of genes key to lipid homeostasis, and this appears to be generally similar in salmon and mammals (Minghetti et al., 2011). SREBP2 is primarily responsible for activation of genes involved in cholesterol synthesis and activate the transcription of mevalonate pathway (cholesterol biosynthesis) genes, such as HMGCR (Liscum, 2008). The positive association between liver fat and SREPB2 expression observed in the current study therefore agrees with the most consistently and highly upregulated group of genes that could be identified here, those of the mevalonate pathway, including HMGCR (Figure 6).

**FIGURE 9:**
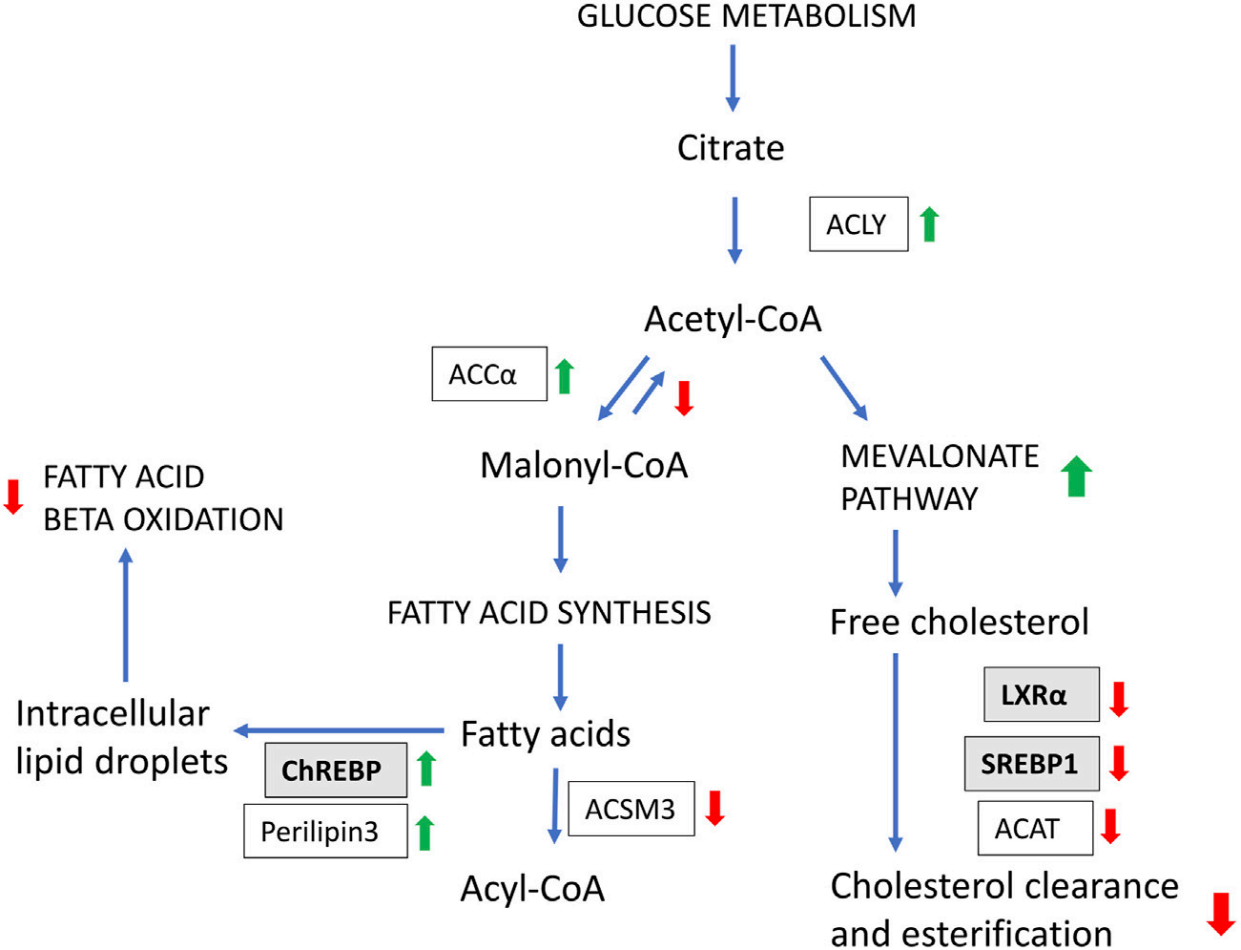
Hypothesis of how metabolic processes are linked with increased liver fat accumulation, based on gene expression results. Boxes indicate genes/enzymes. Colored arrows up and down indicate positive and negative associations with liver fat, respectively. Transcription factors highlighted in grey boxes.

Further to the upregulation of cholesterol biosynthesis genes, the results suggest that fish with high liver fat has reduced clearance of free cholesterol (Figure 9). Under normal conditions, increased intracellular cholesterol induces the transcription of a range of genes (including LXRα and SREBP-1c) that protect cells from cholesterol overload by clearance and esterification of free cholesterol (Ferré and Foufelle, 2010). However, in the current study, gene expression of both LXR and SREPB1 was negatively associated with liver fat content (Figure 6). Further, genes involved in synthesis of bile acids, which are required for cholesterol clearance were negatively associated with liver fat accumulation (24-*hydroxycholesterol 7-alpha-hydroxylase* and *Alpha-methylacyl-CoA racemase;* Figure 6). Additionally, none of the central genes involved in esterification of free cholesterol (e.g., ACAT and LCAT) increased their expression with increasing liver fat. Although we did not measure cholesterol content in livers, these results do suggest that the cholesterol clearance and esterifying process is unable to keep up with the overload of the *de novo* synthesized cholesterol, leading to an unhealthy increase of free cholesterol levels in the liver.

These findings are in accordance with several studies in humans linking cholesterol synthesis and liver fat (Musso et al., 2013). For example, fatty liver was reported to be associated with high cholesterol synthesis and low cholesterol absorption (Simonen et al., 2011). In another study, increased SREBP-2 and HMGCR expression paralleled accumulation of free cholesterol in the liver (Caballero et al., 2009). Further, Puri et al. (2007) showed that there is a stepwise increment in hepatic free cholesterol content from normal livers to NAFLD. Although knowledge is limited in Atlantic salmon, previous studies have shown that replacement of dietary fish oil with vegetable oils results in upregulation of genes of cholesterol biosynthesis, paralleled with increased liver fat accumulation (Leaver et al., 2008; Sanden et al., 2016).

Overall, these results demonstrate a difference in liver function related to cholesterol metabolism between salmon with inherently high and low liver fat accumulation. The mechanisms appear analogous to those observed in mammals, potentially leading to harmful free cholesterol accumulation in salmon with higher liver fat accumulation. These results suggest that liver lipid accumulation in Atlantic salmon is associated with adverse health effects, even when fish are fed a diet relatively high in fish-oil.

### 4.5 Increased conversion of carbohydrates to lipids

The fatty acid analysis of livers showed that the metabolic processes that cause excess liver fat deposition leads to accumulation of 16:1n-7, 18:2n-6 and 18:1n-9. The fatty acids 18:2n-6 and 18:1n-9 are abundant in fish feed, suggesting that excess liver fat may come from feed fatty acids. Additionally, 16:1n-7 and 18:1n-9 are products of *de novo* lipogenesis (DNL), with 16:1n-7 being a primary DNL product. In humans, elevated 16:1n-7 levels in the liver are linked to NAFLD and hepatic lipogenesis rates (Lee et al., 2015). This fatty acid pattern aligns with previous studies on liver fat and health in salmon. Dessen et al. (2020) observed higher 16:1n-7 and 18:1n-9 levels in dying fish compared to survivors during a sudden mortality event of seemingly healthy farmed salmon. Feed trials in Atlantic salmon have also indicated that 18:1n-9 accumulates at the expense of other fatty acids in livers of fish fed low EPA + DHA diets (Hundal et al., 2022; Ruyter et al., 2006). These results suggest increased DNL and/or reduced VLDL secretion in fish with higher liver fat.

The gene expression results regarding DNL were conflicting; fatty acid synthase, the rate-limiting enzyme in the fatty acid synthesis pathway, was not significantly associated with liver fat, while two of the major regulators that promotes DNL, LXRα and SREBP1c, were negatively associated with liver fat. However, expression of other key genes pointed to increased conversion of carbohydrates to lipids (and possibly increased DNL). This included ChREBP, ACLY and ACCα, which were all positively associated with liver fat. (Figure 7). ChREBP activity increases in liver disease, contributing to hepatic steatosis by stimulating the lipogenic pathway (Abdul-Wahed et al., 2017). ACLY links carbohydrate and lipid metabolism, with high expression linked to fatty liver and NAFLD (Chypre et al., 2012; Wang et al., 2009). Further, Expression of ACCα was positively associated with liver fat (Figure 7). ACCα produces malonyl-CoA and is regarded as the pace-setting enzyme for fatty acid synthesis, and is induced by ChREBP (Sul and Smith, 2008). In addition, Malonyl-CoA decarboxylase, which catalyzes the opposite reaction of ACCα, was negatively associated with liver fat (Figure 7).

Carnivorous fish like Atlantic salmon are not well adapted to high dietary carbohydrates, although modern salmon feed contains significant carbohydrates. While key carbohydrate metabolism genes are present, their functions in salmonids are not well understood. However, salmonids can convert carbohydrates to lipids, as shown by Bou et al. (2016), who demonstrated active DNL in Atlantic salmon adipocytes. Selected lines of rainbow trout also showed increased dietary carbohydrate metabolism linked to enhanced liver DNL (Skiba-Cassy et al., 2009). Activation of ChREBP and DNL usually upregulates Stearoyl-CoA desaturase 1 (SCD1), which converts 18:0 and 16:0 into 18:1n-9 and 16:1n-7, respectively (Emken, 1994). Although this study did not find any association between liver fat and SCD expression, the positive association between liver fat and ChREBP expression might explain the elevated 16:1n-7 and 18:1n-9 levels in livers with higher fat content.

### 4.6 Triacylglyceride synthesis

Surprisingly, results indicate lower triacylglyceride (TAG) formation in livers with higher fat levels compared to those with lower fat levels. TAG formation is complex, and regulated by SREBP-1c, PPARγ, and LXR (Hertzel et al., 2008). Although PPARγ was not significantly associated with liver fat levels in this study, the negative association of liver fat with LXRα, SREBP-1, GPAT and DGAT expression suggests lower TAG formation in fattier livers. DGAT enzymes catalyze the final and the only committed step in TAG biosynthesis (Hertzel et al., 2008), and GPAT channels fatty acids into TAG for VLDL secretion, significantly impacting TAG synthesis in mammals (Wendel et al., 2009). Overall, these results do not indicate higher TAG synthesis in fish with higher liver fat levels. However, this does not exclude increased TAG synthesis as the cause of elevated liver fat, as the results might reflect a downregulation in this pathway in fish with already high liver fat levels (negative feedback loop).

### 4.7 Phospholipids

The gene expression results indicated altered phospholipid (PL) composition, especially increased phosphatidylcholine (PC) synthesis in fish with a higher liver fat content. Changes in hepatic PL composition have previously been linked to fatty liver disease (van der Veen et al., 2017). The apparent changes in PC synthesis are especially interesting as essential phospholipids rich in PC is a widely used treatment option for fatty liver disease in humans (Osipova et al., 2022). Because PC biosynthesis is required for normal secretion of very low-density lipoprotein (VLDL) from hepatocytes, some studies suggest that liver TAG accumulation is due to reduced availability of PC (and apolipoprotein B100) (Vance et al., 1997; Watkins et al., 2003). There is limited knowledge in salmonids regarding the role of PC in liver fat accumulation, however, a link between dietary PC and accumulation of lipid droplets in intestinal cells has been observed (Krogdahl et al., 2020).

### 4.8 Reduced beta-oxidation capacity

The results regarding fatty acid beta-oxidation, the breakdown of fatty acids, were somewhat inconsistent, but most genes showed a negative association with liver fat (Figure 7). This suggests that fish with high liver fat have a reduced capacity to oxidize and utilize fatty acids as fuel, potentially contributing to fat accumulation in the liver. Additionally, there were compelling indications of increased inhibition of fatty acid beta-oxidation in livers with higher fat content. Specifically, livers with elevated fat levels demonstrated increased expression of ACLY and ACC (Figure 7), which promotes the formation of Malonyl-CoA. Simultaneously, Malonyl-CoA decarboxylase, responsible for breaking down Malonyl-CoA, was negatively associated with liver fat (Figure 7). Given that Malonyl-CoA acts as an inhibitor of fatty acid beta-oxidation (Sul and Smith, 2008), its increased formation and reduced breakdown point to reduced mitochondrial beta-oxidation activity in livers with higher levels of fat.

Furthermore, the negative correlation between liver fat and gene expression of ACOX1 - the rate-limiting enzyme of peroxisomal beta-oxidation (Figure 7) - strongly indicates that this pathway is downregulated in livers with high lipid content. In addition, LXR–which regulates ACOX1 (Hu et al., 2005) - also showed negative gene expression association with increased liver fat content, supporting this observation. Peroxisomal beta-oxidation is well-established as responsible for oxidizing very-long-chain fatty acids (>C20). The peroxisomal beta-oxidation capacity in salmon liver is high and significantly contributes to total hepatic beta-oxidation (Stubhaug et al., 2007), it is therefore plausible that this pathway is involved in the increased hepatic lipid accumulation observed.

### 4.9 Potential role of the omega-3 fatty acids EPA and DHA in liver fat accumulation

High liver fat coincided with low relative EPA and DHA content in the liver, with a strong negative phenotypic correlation of −0.9 (Figure 3). This agrees with studies that have reported that liver lipids of patients with NAFLD contain less EPA and DHA compared to control subjects (de Castro and Calder, 2018; Scorletti and Byrne, 2018). However, since all fish in the current study were fed the same diet and we only have data from one time-point, it is not possible to separate the effects of low omega-3 and high liver fat. It is also not possible to determine a possible cause-and effect relationship between these two factors. Nonetheless, it is interesting to notice that the metabolic disturbances associated with high liver fat in the current study are similar to those associated with low marine omega-3 levels in the feed (resulting in high liver fat) in a recent study on Atlantic salmon (Hundal et al., 2022). Considering the current knowledge on the influence of omega-3 fatty acids on liver fat, this is a factor worth mentioning and pursuing in future studies. Numerous studies in humans and animal models have reported that dietary EPA and DHA decreased TAG accumulation in liver, probably through decreasing hepatic DNL and promoting beta-oxidation over TAG synthesis (Calder, 2022).

The lower relative EPA and DHA content observed in the fish with higher liver fat can be due to already high lipid levels, which have caused increased utilization and loss of these fatty acids driven by inflammation and oxidative stress. Alternatively, a dilution effect of EPA and DHA has occurred when more FAs from the feed and/or DNL products are deposited in the liver. However, based on the results of the current study we cannot exclude the possibility that the reduction in EPA and DHA is, at least, further promoting or accelerating excess lipid deposition in the liver.

## 5 Conclusion

Liver fat deposition in a population of Atlantic salmon fed a commercial broodstock feed showed large variation, with frequent occurrences of high liver fat accumulation. A heritability estimate of 0.38 and a genetic coefficient of variation of 20% suggest substantial potential for selective breeding to reduce liver fat deposition in Atlantic salmon.

Liver fat deposition in Atlantic salmon is likely a polygenic trait, as no large QTLs were detected by genome-wide association studies. The expression of numerous genes involved in lipid metabolic processes was associated with liver fat content, including key transcription factors regulating lipid metabolism such as LXR, SREBP1, and ChREBP. The results clearly indicated a link between liver fat and increased cholesterol synthesis in Atlantic salmon, similar to observations in humans, with potentially harmful accumulation of free cholesterol.

Further, the gene expression results pointed to reduced peroxisomal fatty acid β-oxidation, increased conversion of carbohydrates to lipids, and possibly increased *de novo* lipogenesis, resulting in the accumulation of 18:1 n-9 and 16:1 n-7 fatty acids. It remains unclear which of these results are consequences of high fat levels and which are underlying metabolic differences causing higher liver fat levels in certain individuals. Nonetheless, the results provide new insights into the metabolic profile of livers in fish with inherent differences in liver fat content.

## Data Availability

The raw data supporting the conclusions of this article will be made available by the authors, without undue reservation.

## References

[B1] Abdul-Wahed A. Guilmeau S. Postic C. (2017). Sweet sixteenth for ChREBP: established roles and future goals. Cell Metab. 26 (2), 324–341. 10.1016/j.cmet.2017.07.004 28768172

[B2] Alves-Bezerra M. Cohen D. E. (2017). Triglyceride metabolism in the liver. Compr. Physiol. 8 (1), 1–8. 10.1002/cphy.c170012 29357123 PMC6376873

[B3] Anders S. Huber W. (2010). Differential expression analysis for sequence count data. Genome Biol. 11 (10), R106. 10.1186/gb-2010-11-10-r106 20979621 PMC3218662

[B4] Aursand M. Bleivik B. Rainuzzo J. R. Jorgensen L. Mohr V. (1994). Lipid distribution and composition of commercially farmed Atlantic Salmon (*Salmo salar*). J. Sci. Food Agric. 64 (2), 239–248. 10.1002/jsfa.2740640214

[B5] Banini B. A. Sanyal A. J. (2016). Nonalcoholic fatty liver disease: epidemiology, pathogenesis, natural history, diagnosis, and current treatment options. Clin. Med. Insights. Ther. 8, 75–84. 10.4137/cmt.s18885 28670148 PMC5491796

[B6] Bou M. Berge G. M. Baeverfjord G. Sigholt T. Østbye T.-K. Ruyter B. (2017). Low levels of very-long-chain n-3 PUFA in Atlantic salmon (*Salmo salar*) diet reduce fish robustness under challenging conditions in sea cages. J. Nutr. Sci. 6, e32. 10.1017/jns.2017.28 29152236 PMC5672314

[B7] Bou M. Todorčević M. Torgersen J. Škugor S. Navarro I. Ruyter B. (2016). *De novo* lipogenesis in Atlantic salmon adipocytes. Biochimica Biophysica Acta - General Subj. 1860 (1), 86–96. 10.1016/j.bbagen.2015.10.022 26518346

[B8] Bransden M. P. Carter C. G. Nichols P. D. (2003). Replacement of fish oil with sunflower oil in feeds for Atlantic salmon (*Salmo salar* L.): effect on growth performance, tissue fatty acid composition and disease resistance. Comp. Biochem. Physiology Part B Biochem. Mol. Biol. 135 (4), 611–625. 10.1016/s1096-4959(03)00143-x 12892753

[B9] Caballero F. Fernández A. De Lacy A. M. Fernández-Checa J. C. Caballería J. García-Ruiz C. (2009). Enhanced free cholesterol, SREBP-2 and StAR expression in human NASH. J. Hepatology 50 (4), 789–796. 10.1016/j.jhep.2008.12.016 19231010

[B10] Calder P. C. (2022). Omega-3 fatty acids and metabolic partitioning of fatty acids within the liver in the context of nonalcoholic fatty liver disease. Curr. Opin. Clin. Nutr. Metab. Care 25 (4), 248–255. 10.1097/MCO.0000000000000845 35762160

[B11] Chypre M. Zaidi N. Smans K. (2012). ATP-citrate lyase: a mini-review. Biochem. Biophysical Res. Commun. 422 (1), 1–4. 10.1016/j.bbrc.2012.04.144 22575446

[B12] Cruz-Garcia L. Minghetti M. Navarro I. Tocher D. R. (2009). Molecular cloning, tissue expression and regulation of liver X Receptor (LXR) transcription factors of Atlantic salmon (*Salmo salar*) and rainbow trout (Oncorhynchus mykiss). Comp. Biochem. Physiology Part B Biochem. Mol. Biol. 153 (1), 81–88. 10.1016/j.cbpb.2009.02.001 19416695

[B13] de Castro G. S. Calder P. C. (2018). Non-alcoholic fatty liver disease and its treatment with n-3 polyunsaturated fatty acids. Clin. Nutr. 37 (1), 37–55. 10.1016/j.clnu.2017.01.006 28139281

[B14] Dessen J. E. Østbye T. K. Ruyter B. Bou M. Thomassen M. S. Rørvik K. A. (2020). Sudden increased mortality in large seemingly healthy farmed Atlantic salmon (Salmo salar L.) was associated with environmental and dietary changes. J. Appl. Aquac., 1–18. 10.1080/10454438.2020.1726237

[B15] Emken E. A. (1994). Metabolism of dietary stearic acid relative to other fatty acids in human subjects. Am. J. Clin. Nutr. 60 (6 Suppl. l), 1023S-1028S–1028s. 10.1093/ajcn/60.6.1023S 7977144

[B16] Eslam M. Valenti L. Romeo S. (2018). Genetics and epigenetics of NAFLD and NASH: clinical impact. J. Hepatol. 68 (2), 268–279. 10.1016/j.jhep.2017.09.003 29122391

[B17] Espe M. Xie S. Chen S. Pedro A. Holen E. (2019). Development of a fatty liver model using oleic acid in primary liver cells isolated from Atlantic salmon and the prevention of lipid accumulation using metformin. Aquac. Nutr. 25 (3), 737–746. 10.1111/anu.12905

[B18] Feng J. Qiu S. Zhou S. Tan Y. Bai Y. Cao H. (2022). mTOR: a potential new target in nonalcoholic fatty liver disease. Int. J. Mol. Sci. 23 (16), 9196. 10.3390/ijms23169196 36012464 PMC9409235

[B19] Ferré P. Foufelle F. (2010). Hepatic steatosis: a role for *de novo* lipogenesis and the transcription factor SREBP-1c. Diabetes, Obes. Metabolism 12 (s2), 83–92. 10.1111/j.1463-1326.2010.01275.x 21029304

[B20] Folch J. Lees M. Stanley G. H. S. (1957). A Simple method for the isolation and purification of total lipides from animal tissues. J. Biol. Chem. 226 (1), 497–509. 10.1016/s0021-9258(18)64849-5 13428781

[B21] Gjedrem T. (2005). Selection and breeding programs in aquaculture, Springer.

[B22] Hazel L. N. (1943). The genetic basis for constructing selection indexes. Genetics 28 (6), 476–490. 10.1093/genetics/28.6.476 17247099 PMC1209225

[B23] Heeren J. Scheja L. (2021). Metabolic-associated fatty liver disease and lipoprotein metabolism. Mol. Metab. 50, 101238. 10.1016/j.molmet.2021.101238 33892169 PMC8324684

[B24] Hertzel A. V. Thompson B. R. Wiczer B. M. Bernlohr D. A. (2008). “CHAPTER 10 - lipid metabolism in adipose tissue,” in Biochemistry of lipids, lipoproteins and membranes. Editors Vance D. E. Vance J. E. Fifth Edition (San Diego: Elsevier), 277–304.

[B25] Horn S. S. Sonesson A. K. Krasnov A. Moghadam H. Hillestad B. Meuwissen T. H. E. (2019). Individual differences in EPA and DHA content of Atlantic salmon are associated with gene expression of key metabolic processes. Sci. Rep. 9 (1), 3889. 10.1038/s41598-019-40391-2 30846825 PMC6405848

[B26] Hu T. Foxworthy P. Siesky A. Ficorilli J. V. Gao H. Li S. (2005). Hepatic peroxisomal fatty acid beta-oxidation is regulated by liver X receptor alpha. Endocrinology 146 (12), 5380–5387. 10.1210/en.2005-0591 16123164

[B27] Hundal B. K. Lutfi E. Sigholt T. Rosenlund G. Liland N. S. Glencross B. (2022). A piece of the puzzle—possible mechanisms for why low dietary EPA and DHA cause hepatic lipid accumulation in atlantic salmon (*Salmo salar*). Metabolites 12 (2), 159. 10.3390/metabo12020159 35208233 PMC8877222

[B28] Karasawa K. Tanigawa K. Harada A. Yamashita A. (2019). Transcriptional regulation of acyl-CoA:glycerol-sn-3-phosphate acyltransferases. Int. J. Mol. Sci. 20 (4), 964. 10.3390/ijms20040964 30813330 PMC6412627

[B29] Kjaer M. A. Vegusdal A. Gjøen T. Rustan A. C. Todorcević M. Ruyter B. (2008). Effect of rapeseed oil and dietary n-3 fatty acids on triacylglycerol synthesis and secretion in Atlantic salmon hepatocytes. Biochim. Biophys. Acta 1781 (3), 112–122. 10.1016/j.bbalip.2007.12.004 18222184

[B30] Kjær M. A. Todorčević M. Torstensen B. E. Vegusdal A. Ruyter B. (2008). Dietary n-3 HUFA affects mitochondrial fatty acid beta-oxidation capacity and susceptibility to oxidative stress in Atlantic salmon. Lipids 43 (9), 813–827. 10.1007/s11745-008-3208-z 18615261

[B31] Krogdahl Å. Hansen A. K. G. Kortner T. M. Bjӧrkhem I. Krasnov A. Berge G. M. (2020). Choline and phosphatidylcholine, but not methionine, cysteine, taurine and taurocholate, eliminate excessive gut mucosal lipid accumulation in Atlantic salmon (*Salmo salar* L). Aquaculture 528, 735552. 10.1016/j.aquaculture.2020.735552

[B32] Leaver M. J. Villeneuve L. A. N. Obach A. Jensen L. Bron J. E. Tocher D. R. (2008). Functional genomics reveals increases in cholesterol biosynthetic genes and highly unsaturated fatty acid biosynthesis after dietary substitution of fish oil with vegetable oils in Atlantic salmon (*Salmo salar*). Bmc Genomics 9, 299. 10.1186/1471-2164-9-299 18577222 PMC2459193

[B33] Lee J. J. Lambert J. E. Hovhannisyan Y. Ramos-Roman M. A. Trombold J. R. Wagner D. A. (2015). Palmitoleic acid is elevated in fatty liver disease and reflects hepatic lipogenesis. Am. J. Clin. Nutr. 101 (1), 34–43. 10.3945/ajcn.114.092262 25527748 PMC4266891

[B34] Lee S. H. Yang J. Goddard M. E. Visscher P. M. Wray N. R. (2012). Estimation of pleiotropy between complex diseases using single-nucleotide polymorphism-derived genomic relationships and restricted maximum likelihood. Bioinforma. Oxf. Engl. 28 (19), 2540–2542. 10.1093/bioinformatics/bts474 PMC3463125 22843982

[B35] Liang M. J. Wang Z. P. Xu L. Leng L. Wang S. Z. Luan P. (2015). Estimating the genetic parameters for liver fat traits in broiler lines divergently selected for abdominal fat. Genet. Mol. Res. 14 (3), 9646–9654. 10.4238/2015.August.14.27 26345897

[B36] Liscum L. (2008). “CHAPTER 14 - cholesterol biosynthesis,” in Biochemistry of lipids, lipoproteins and membranes. Editors Vance D. E. Vance J. E. Fifth Edition (San Diego: Elsevier), 399–421.

[B37] Marie-Etancelin C. Vitezica Z. G. Bonnal L. Fernandez X. Bastianelli D. (2014). Selecting the quality of mule duck fatty liver based on near-infrared spectroscopy. Genet. Sel. Evol. 46 (1), 38. 10.1186/1297-9686-46-38 24917150 PMC4078935

[B38] Mason M. E. Waller G. R. (1964). Dimethoxypropane induced transesterification of fats + oils in preparation of methyl esters for gas chromatographic analysis. Anal. Chem. 36 (3), 583–and. 10.1021/ac60209a008

[B39] Minghetti M. Leaver M. J. Tocher D. R. (2011). Transcriptional control mechanisms of genes of lipid and fatty acid metabolism in the Atlantic salmon (*Salmo salar* L.) established cell line, SHK-1. Biochimica Biophysica Acta (BBA) - Mol. Cell Biol. Lipids 1811 (3), 194–202. 10.1016/j.bbalip.2010.12.008 21193059

[B40] Minkina O. Cheverud J. M. Fawcett G. Semenkovich C. F. Kenney-Hunt J. P. (2012). Quantitative trait loci affecting liver fat content in mice. G3 Genes|Genomes|Genetics 2 (9), 1019–1025. 10.1534/g3.112.003343 22973538 PMC3429915

[B41] Moghadam H. K. Johnsen H. Robinson N. Andersen O. Jorgensen E. H. Johnsen H. K. (2017). Impacts of early life stress on the methylome and transcriptome of atlantic salmon. Sci. Rep. 7, 5023. 10.1038/s41598-017-05222-2 28694447 PMC5504078

[B42] Morais S. Pratoomyot J. Taggart J. B. Bron J. E. Guy D. R. Bell J. G. (2011a). Genotype-specific responses in Atlantic salmon (*Salmo salar*) subject to dietary fish oil replacement by vegetable oil: a liver transcriptomic analysis. BMC genomics 12, 255. 10.1186/1471-2164-12-255 21599965 PMC3113789

[B43] Morais S. Pratoomyot J. Torstensen B. E. Taggart J. B. Guy D. R. Gordon Bell J. (2011b). Diet × genotype interactions in hepatic cholesterol and lipoprotein metabolism in Atlantic salmon (*Salmo salar*) in response to replacement of dietary fish oil with vegetable oil. Br. J. Nutr. 106 (10), 1457–1469. 10.1017/S0007114511001954 21736795

[B44] Mørkøre T. Å. Magnus D. Sanden K. W. Bjerke M. T. Rørvik K. G. (2012). Tekstur og fett i laksefilet. Nofima Rapp. Nofima AS.

[B45] Moya-Falcon C. Thomassen M. S. Jakobsen J. V. Ruyter B. (2005). Effects of dietary supplementation of rapeseed oil on metabolism of [1-14C]18:1n-9, [1-14C]20:3n-6, and [1-14C]20:4n-3 in Atlantic salmon hepatocytes. Lipids 40 (7), 709–717. 10.1007/s11745-005-1434-9 16196422

[B46] Mulder H. Veerkamp R. Ducro B. Van Arendonk J. Bijma P. (2006). Optimization of dairy cattle breeding programs for different environments with genotype by environment interaction. J. dairy Sci. 89 (5), 1740–1752. 10.3168/jds.S0022-0302(06)72242-1 16606745

[B47] Musso G. Gambino R. Cassader M. (2013). Cholesterol metabolism and the pathogenesis of non-alcoholic steatohepatitis. Prog. Lipid Res. 52 (1), 175–191. 10.1016/j.plipres.2012.11.002 23206728

[B48] Namjou B. Lingren T. Huang Y. Parameswaran S. Cobb B. L. Stanaway I. B. (2019). GWAS and enrichment analyses of non-alcoholic fatty liver disease identify new trait-associated genes and pathways across eMERGE Network. BMC Med. 17 (1), 135. 10.1186/s12916-019-1364-z 31311600 PMC6636057

[B49] Ortega S. Lutfi E. Horn S. S. Durland E. (2024). Quantification of fat content in the liver of different aquaculture fish species using hyperspectral image analysis *[Manuscript submitted for publication]*. Unpublished manuscript.

[B50] Osipova D. Kokoreva K. Lazebnik L. Golovanova E. Pavlov C. Dukhanin A. (2022). Regression of liver steatosis following phosphatidylcholine administration: a review of molecular and metabolic pathways involved. Front. Pharmacol. 13, 797923. 10.3389/fphar.2022.797923 35359878 PMC8960636

[B51] Puri P. Baillie R. A. Wiest M. M. Mirshahi F. Choudhury J. Cheung O. (2007). A lipidomic analysis of nonalcoholic fatty liver disease. Hepatology 46 (4), 1081–1090. 10.1002/hep.21763 17654743

[B52] Robertson A. (1959). The sampling variance of the genetic correlation coefficient. Biometrics 15 (3), 469–485. 10.2307/2527750

[B53] Romeo S. Kozlitina J. Xing C. Pertsemlidis A. Cox D. Pennacchio L. A. (2008). Genetic variation in PNPLA3 confers susceptibility to nonalcoholic fatty liver disease. Nat. Genet. 40 (12), 1461–1465. 10.1038/ng.257 18820647 PMC2597056

[B54] Ruyter B. Moya-Falcon C. Rosenlund G. Vegusdal A. (2006). Fat content and morphology of liver and intestine of Atlantic salmon (*Salmo salar*): effects of temperature and dietary soybean oil. Aquaculture 252 (2-4), 441–452. 10.1016/j.aquaculture.2005.07.014

[B55] Ruyter B. Thomassen M. S. (1999). Metabolism of n-3 and n-6 fatty acids in Atlantic salmon liver: stimulation by essential fatty acid deficiency. Lipids 34 (11), 1167–1176. 10.1007/s11745-999-0468-3 10606039

[B56] Sanden M. Liland N. S. Sæle Ø. Rosenlund G. Du S. Torstensen B. E. (2016). Minor lipid metabolic perturbations in the liver of Atlantic salmon (*Salmo salar* L.) caused by suboptimal dietary content of nutrients from fish oil. Fish Physiology Biochem. 42 (5), 1463–1480. 10.1007/s10695-016-0233-3 27154233

[B57] Schulz H. (2008). “CHAPTER 5 - oxidation of fatty acids in eukaryotes,” in Biochemistry of lipids, lipoproteins and membranes. Editors Vance D. E. Vance J. E. Fifth Edition (San Diego: Elsevier), 131–154.

[B58] Scorletti E. Byrne C. D. (2018). Omega-3 fatty acids and non-alcoholic fatty liver disease: evidence of efficacy and mechanism of action. Mol. Asp. Med. 64, 135–146. 10.1016/j.mam.2018.03.001 29544992

[B59] Seo M. Kim K. Yoon J. Jeong J. Y. Lee H.-J. Cho S. (2016). RNA-seq analysis for detecting quantitative trait-associated genes. Sci. Rep. 6, 24375. 10.1038/srep24375 27071914 PMC4829873

[B60] Simonen P. Kotronen A. Hallikainen M. Sevastianova K. Makkonen J. Hakkarainen A. (2011). Cholesterol synthesis is increased and absorption decreased in non-alcoholic fatty liver disease independent of obesity. J. Hepatology 54 (1), 153–159. 10.1016/j.jhep.2010.05.037 20947198

[B61] Skiba-Cassy S. Lansard M. Panserat S. Médale F. (2009). Rainbow trout genetically selected for greater muscle fat content display increased activation of liver TOR signaling and lipogenic gene expression. Am. J. Physiology-Regulatory, Integr. Comp. Physiology 297 (5), R1421–R1429. 10.1152/ajpregu.00312.2009 19710390

[B62] Sookoian S. Pirola C. J. (2017). Genetic predisposition in nonalcoholic fatty liver disease. Clin. Mol. hepatology 23 (1), 1–12. 10.3350/cmh.2016.0109 PMC5381829 28268262

[B63] Stubhaug I. Froyland L. Torstensen B. E. (2005). beta-oxidation capacity of red and white muscle and liver in Atlantic salmon (*Salmo salar* L.) - effects of increasing dietary rapeseed oil and olive oil to replace capelin oil. Lipids 40 (1), 39–47. 10.1007/s11745-005-1358-4 15825829

[B64] Stubhaug I. Lie O. Torstensen B. E. (2007). Fatty acid productive value and beta-oxidation capacity in Atlantic salmon (*Salmo salar* L.) fed on different lipid sources along the whole growth period. Aquac. Nutr. 13 (2), 145–155. 10.1111/j.1365-2095.2007.00462.x

[B65] Sul H. S. Smith S. (2008). “CHAPTER 6 - fatty acid synthesis in eukaryotes,” in Biochemistry of lipids, lipoproteins and membranes. Editors Vance D. E. Vance J. E. Fifth Edition (San Diego: Elsevier), 155–190.

[B66] Torstensen B. E. Espe M. Stubhaug I. Lie O. (2011). Dietary plant proteins and vegetable oil blends increase adiposity and plasma lipids in Atlantic salmon (*Salmo salar* L.). Br. J. Nutr. 106 (5), 633–647. 10.1017/S0007114511000729 21535902

[B67] Trapnell C. Williams B. A. Pertea G. Mortazavi A. Kwan G. van Baren M. J. (2010). Transcript assembly and quantification by RNA-Seq reveals unannotated transcripts and isoform switching during cell differentiation. Nat. Biotechnol. 28 (5), 511–515. 10.1038/nbt.1621 20436464 PMC3146043

[B68] Vance D. E. Walkey C. J. Cui Z. (1997). Phosphatidylethanolamine N-methyltransferase from liver. Biochimica Biophysica Acta (BBA) - Lipids Lipid Metabolism 1348 (1), 142–150. 10.1016/s0005-2760(97)00108-2 9370326

[B69] van der Veen J. N. Kennelly J. P. Wan S. Vance J. E. Vance D. E. Jacobs R. L. (2017). The critical role of phosphatidylcholine and phosphatidylethanolamine metabolism in health and disease. Biochimica Biophysica Acta (BBA) - Biomembr. 1859 (9), 1558–1572. 10.1016/j.bbamem.2017.04.006 28411170

[B70] Vegusdal A. Gjøen T. Berge R. K. Thomassen M. S. Ruyter B. (2005). Effect of 18∶1n−9, 20∶5n−3, and 22∶6n−3 on lipid accumulation and secretion by atlantic salmon hepatocytes. Lipids 40 (5), 477–486. 10.1007/s11745-005-1407-z 16094857

[B71] Wang Q. Jiang L. Wang J. Li S. Yu Y. You J. (2009). Abrogation of hepatic ATP-citrate lyase protects against fatty liver and ameliorates hyperglycemia in leptin receptor-deficient mice. Hepatology 49 (4), 1166–1175. 10.1002/hep.22774 19177596

[B72] Wang Q. Li D. Zhu J. Zhang M. Zhang H. Cao G. (2020). Perforin acts as an immune regulator to prevent the progression of NAFLD. Front. Immunol. 11, 846. 10.3389/fimmu.2020.00846 32528465 PMC7256195

[B73] Watkins S. M. Zhu X. Zeisel S. H. (2003). Phosphatidylethanolamine-N-methyltransferase activity and dietary choline regulate liver-plasma lipid flux and essential fatty acid metabolism in mice. J. Nutr. 133 (11), 3386–3391. 10.1093/jn/133.11.3386 14608048

[B74] Wendel A. A. Lewin T. M. Coleman R. A. (2009). Glycerol-3-phosphate acyltransferases: rate limiting enzymes of triacylglycerol biosynthesis. Biochimica Biophysica Acta (BBA) - Mol. Cell Biol. Lipids 1791 (6), 501–506. 10.1016/j.bbalip.2008.10.010 PMC2737689 19038363

[B75] Xia B. Cai G. H. Yang H. Wang S. P. Mitchell G. A. Wu J. W. (2017). Adipose tissue deficiency of hormone-sensitive lipase causes fatty liver in mice. PLOS Genet. 13 (12), e1007110. 10.1371/journal.pgen.1007110 29232702 PMC5741266

[B76] Yang J. Zaitlen N. A. Goddard M. E. Visscher P. M. Price A. L. (2014). Advantages and pitfalls in the application of mixed-model association methods. Nat. Genet. 46 (2), 100–106. 10.1038/ng.2876 24473328 PMC3989144

