# Supplementary material for: Genetic and metabolic characterization of individual differences in liver fat accumulation in Atlantic salmon
Source: Front Genet. 2025 Feb 13;16:1512769. doi: 10.3389/fgene.2025.1512769 (PMC11865213; doi:10.3389/fgene.2025.1512769)
Supplement: Supplementary file 4 [file Table3.docx]

Table S1. Top 10 SNPs with their identified candidate genes. Position physical position of SNP in base pairs, A1 & A2 minor & major alleles, respectively, MAF minor allele frequency, α Allele substitution effect, SE standard error, P significance value.

| **Chr** | **SNP** | **Position** | **A1** | **A2** | **MAF** | **α** | **SE** | **p-value** | **Candidate gene name** | **Location** |
| --- | --- | --- | --- | --- | --- | --- | --- | --- | --- | --- |
| ssa15 | AX-87183264 | 72071045 | A | B | 0.084 | 1.407 | 0.314 | 7.23E-06 | mTOR | 71706074-71838947 |
| ssa23 | AX-98317599 | 16469836 | B | A | 0.298 | 0.753 | 0.172 | 1.17E-05 |  |  |
| ssa21 | AX-87924296 | 13929700 | B | A | 0.366 | 0.661 | 0.163 | 5.00E-05 | glycerol-3-phosphate dehydrogenase | 14051211-14100791 |
| ssa16 | AX-87098707 | 9532406 | A | B | 0.003 | 4.950 | 1.227 | 5.50E-05 |  |  |
| ssa18 | AX-97896826 | 65948775 | A | B | 0.477 | 0.620 | 0.157 | 8.32E-05 | perforin 1 | 66237619-66242891 |
| ssa05 | AX-88055452 | 70043625 | A | B | 0.114 | 1.021 | 0.261 | 9.08E-05 | Hormone-sensitive lipase | 69985959-70027850 |
| ssa23 | AX-88142019 | 27166373 | A | B | 0.305 | -0.693 | 0.177 | 9.15E-05 |  |  |
| ssa18 | AX-87765492 | 64703505 | A | B | 0.185 | 0.809 | 0.208 | 9.82E-05 |  |  |
| ssa23 | AX-87913438 | 18700581 | A | B | 0.484 | -0.638 | 0.167 | 0.000127 |  |  |
| ssa18 | AX-97870866 | 64700815 | A | B | 0.181 | 0.796 | 0.209 | 0.000136 |  |  |
